# Supplementary material for: Severe drought exposure in utero associates to children’s epigenetic age acceleration in a global climate change hot spot
Source: Nat Commun. 2024 May 16;15:4140. doi: 10.1038/s41467-024-48426-7 (PMC11099019; doi:10.1038/s41467-024-48426-7)
Supplement: Supplementary file 1 — Supplementary Information [file 41467_2024_48426_MOESM1_ESM.pdf]

Supplementary Table 1: Regression Coefficients from Measure of Epigenetic Age Accelerations on Drought Exposure

	Intercept	Drought_ Exposed	Sex_ Female	Epi	Fib	Gravida	Birth Season Wet
EAA_{PedBE}[a]	-1.06* (-1.75, -0.34)	0.11 (-0.11, 0.31)[b]	0.03 (-0.25, 0.31)	0.98 (-0.08, 2.05)	26.35* (13.98, 39.62)	0.04 (-0.03, 0.1)	-0.04 (-0.26, 0.18)
EAA_{Wu}	-0.52 (-1.59, 0.61)	-0.02 (-0.37, 0.33)	0.09 (-0.33, 0.5)	1.92* (0.24, 3.64)	-3.09 (-24.07, 17.69)	0.03 (-0.07, 0.12)	-0.12 (-0.47, 0.24)
$EAA_{Horvath}$	2.24* (0.75, 3.82)	-0.31 (-0.81, 0.18)	0.53 (-0.05, 1.1)	-5.5* (-7.99, -3.16)	-65.69* (-94.8, -35.88)	0.07 (-0.07, 0.2)	-0.48 (-0.98, 0.04)
$EAA_{Skin\&Blood}$	0.3 (-0.45, 1.01)	-0.09 (-0.33, 0.12)	0.19 (-0.1, 0.47)	-1.89* (-2.95, -0.76)	-2.51 (-15.6, 10.85)	-0.01 (-0.07, 0.06)	-0.04 (-0.27, 0.19)
EAA_{Hannum}	-5.61* (-7.46, -3.74)	1.34* (0.74, 1.96)	0.01 (-0.71, 0.69)	21.33* (18.48, 24.31)	-14.37 (-50.62, 21.31)	0.06 (-0.11, 0.22)	0.13 (-0.48, 0.75)
$EAA_{PhenoAge}$	-3.41 (-7.35, 0.59)	0.78 (-0.59, 2.15)	2.41* (1.02, 3.72)	14.76* (8.7, 21.13)	-184.38* (-264.77, -106.01)	0.08 (-0.23, 0.4)	-0.06 (-1.44, 1.23)
$EAA_{GirmAge2}$	-4.9* (-7.07, -2.7)	1.31* (0.61, 1.97)	-0.29 (-1.17, 0.49)	22.65* (19.2, 25.84)	-32.92 (-75.62, 6.35)	0.01 (-0.18, 0.2)	0.17 (-0.55, 0.84)
EAA_{DNAmTL}	0.58* (0.21, 0.97)	-0.13* (-0.24, -0.04)	0.03 (-0.14, 0.19)	-2.27* (-2.81, -1.76)	-0.73 (-6.96, 5.08)	-0.01 (-0.04, 0.03)	0.01 (-0.09, 0.12)
DunedinPACE	1.13* (0.74, 1.49)	-0.03 (-0.13, 0.07)	0 (-0.16, 0.16)	0.79* (0.28, 1.31)	-0.36 (-6.27, 5.76)	0 (-0.03, 0.04)	0.02 (-0.08, 0.13)
DunedinPoAm38	1.3* (0.92, 1.66)	0.01 (-0.09, 0.1)	0 (-0.16, 0.16)	-0.45 (-0.96, 0.04)	-2.31 (-8.3, 3.52)	0 (-0.03, 0.04)	0.01 (-0.09, 0.11)

[a] EAA_{PedBE} denotes the epigenetic age acceleration of PedBE clock. [b] Posterior mean of regression coefficient of drought on age acceleration of PedBE is 0.11, with 95% highest posterior density (HPD) of (-0.11, 0.31). *95% HPD interval not containing zero will be considered significant.

Supplementary Table 2: Regression Coefficients of Drought Exposure in the Univariate Regression Models[a]

	Estimate	Standard Error	Degree of Freedom	t-value	p-value	Adjusted P-value[b]
EAA_{PedBE}	0.09[c]	0.10	151.80	0.90	0.37	0.46
EAA_{Wu}	-0.01	0.18	144.18	-0.06	0.95	0.95
$EAA_{Horvath}$	-0.34	0.25	151.71	-1.37	0.17	0.35
$EAA_{Skin\&Blood}$	-0.10	0.11	143.80	-0.99	0.32	0.46
EAA_{Hannum}	1.35	0.31	147.06	4.38	0.00	0.00*
$EAA_{PhenoAge}$	0.73	0.67	144.26	1.09	0.28	0.46
$EAA_{GirmAge2}$	1.28	0.34	151.06	3.81	0.00	0.00*
EAA_{DNAmTL}	-0.13	0.02	155.14	-8.60	0.00	0.00*
DunedinPACE	-0.03	0.01	148.31	-2.94	0.00	0.01*
DunedinPoAm38	0.00	0.01	145.66	0.65	0.52	0.57

[a]The univariate regression models measure the association between the corresponding epigenetic age acceleration response variable and drought exposure using frequentist approach, adjusting sex, cell type, gravida, and birth season. Each row represents a separate univariate model. P-values were obtained by two-sided t-tests. [b]P-values are adjusted using Benjamini-Hochberg (FDR) method.[1]*Adjusted p-value < 0.05. [c]Estimated regression coefficient of drought exposure on the age acceleration of PedBE is 0.09.

[1] Benjamini, Y., & Hochberg, Y. (1995). Controlling the False Discovery Rate: A Practical and Powerful Approach to Multiple Testing. Journal of the Royal Statistical Society. Series B (Methodological), 57(1), 289–300. http://www.jstor.org/stable/2346101
