# Supplementary material for: Severe drought exposure in utero associates to children’s epigenetic age acceleration in a global climate change hot spot
Source: Nat Commun. 2024 May 16;15:4140. doi: 10.1038/s41467-024-48426-7 (PMC11099019; doi:10.1038/s41467-024-48426-7)
Supplement: Supplementary file 3 — Reporting Summary [file 41467_2024_48426_MOESM3_ESM.pdf]

nature portfolio

Corresponding author(s): Bilinda Straight

Last updated by author(s): 2024-4-16

Reporting Summary

Nature Portfolio wishes to improve the reproducibility of the work that we publish. This form provides structure for consistency and transparency in reporting. For further information on Nature Portfolio policies, see our Editorial Policies and the Editorial Policy Checklist.

Statistics

For all statistical analyses, confirm that the following items are present in the figure legend, table legend, main text, or Methods section.

n/a	Confirmed	
☐	☒	The exact sample size (*n*) for each experimental group/condition, given as a discrete number and unit of measurement
☐	☒	A statement on whether measurements were taken from distinct samples or whether the same sample was measured repeatedly
☐	☒	The statistical test(s) used AND whether they are one- or two-sided<br>*Only common tests should be described solely by name; describe more complex techniques in the Methods section.*
☐	☒	A description of all covariates tested
☐	☒	A description of any assumptions or corrections, such as tests of normality and adjustment for multiple comparisons
☐	☒	A full description of the statistical parameters including central tendency (e.g. means) or other basic estimates (e.g. regression coefficient) AND variation (e.g. standard deviation) or associated estimates of uncertainty (e.g. confidence intervals)
☐	☒	For null hypothesis testing, the test statistic (e.g. *F*, *t*, *r*) with confidence intervals, effect sizes, degrees of freedom and *P* value noted<br>*Give P values as exact values whenever suitable.*
☐	☒	For Bayesian analysis, information on the choice of priors and Markov chain Monte Carlo settings
☐	☒	For hierarchical and complex designs, identification of the appropriate level for tests and full reporting of outcomes
☐	☒	Estimates of effect sizes (e.g. Cohen's *d*, Pearson's *r*), indicating how they were calculated

Our web collection on statistics for biologists contains articles on many of the points above.

Software and code

Policy information about availability of computer code

Data collection	• Epigenetic clocks were derived using R package methylCIPHER from MorganLevineLab, R package dnaMethyAge from Github, R function derived from original clock paper, and R code provided by the authors of “DNA methylation GrimAge version 2”<br>• Cell type proportions were estimated using R package EpiDISH.
Data analysis	• Figures 1 and 3 were generated using Microsoft Excel for Mac Version 16.83.<br>• Figure 4 was generated using R packages ggcorrplot and patchwork.<br>• All code is run in R version 4.1.2<br>• Statistical analyses were conducted using the following packages: MCMCglmm, lmerTest and lme4.<br>• R code for data analyses is available at https://github.com/DuyNgoStats/BayesianEAA.

For manuscripts utilizing custom algorithms or software that are central to the research but not yet described in published literature, software must be made available to editors and reviewers. We strongly encourage code deposition in a community repository (e.g. GitHub). See the Nature Portfolio guidelines for submitting code & software for further information.

Data

Policy information about availability of data

All manuscripts must include a data availability statement. This statement should provide the following information, where applicable:

- Accession codes, unique identifiers, or web links for publicly available datasets
- A description of any restrictions on data availability
- For clinical datasets or third party data, please ensure that the statement adheres to our policy

The source data for all Figures and Tables, including data used to support the findings of this study are supplied with this paper in the Source Data file. Rainfall variables for Figure 1 were calculated from high resolution publicly available historical data (CHIRPS) on the Famine Early Warning Systems Network (FEWSNET: https://earlywarning.usgs.gov/fews). DNA methylation data are based on the Illumina MethylataionEPIC (EPIC) BeadChip array-based platform. The individual-level DNA methylation data and the CHIRPS individual-level location and pregnancy-timed data (used for illustration purposes in Figure 1, not for data analyses) are available under restricted access due to privacy and ethical restrictions because the research partners of this study are a vulnerable group of African indigenous peoples. Access can be obtained by contacting the corresponding author (bilinda.straight@wmich.edu) as follows: Queries for access to verify results will receive a response within 2 weeks, and access will be granted as immediately as possible after approval from the Western Michigan University IRB, but in no more than 2 weeks from approval. Access for new studies will receive a response within 2 weeks and will be subject to restrictions imposed by Kenya's National Commission for Science, Technology, and Innovation (NACOSTI) and the Indigenous community.

Research involving human participants, their data, or biological material

Policy information about studies with human participants or human data. See also policy information about sex, gender (identity/presentation), and sexual orientation and race, ethnicity and racism.

Reporting on sex and gender	The study's used biological sex assigned at birth as reported by parents and recorded in vaccination records, and consistent with Samburu cultural categories. The study design is maternal and same-sex sibling pair design (e.g., control siblings matched drought-exposed children; some families had more than one control sibling). All mothers identified as female based on sex assigned at birth and were the biological mothers of the children. Mothers (female) N = 104; Children, male = 98; female = 115. Total Child N = 213. Children’s ages (1.81 - 9.61 years) were documented with vaccination records and birth certificates and calculated to age in years (minimum of two decimal points). Sex of all participating children as identified by parents and children matched sex reported in health records.
Reporting on race, ethnicity, or other socially relevant groupings	The study recruited Samburu pastoralists, all of whom self-identify as Samburu ethnicity, East Africans in northern Kenya.
Population characteristics	Samburu pastoralists are subsistence livestock herders living primarily in Samburu County, with some also living in adjacent Laikipia County. They engage in a climate change vulnerable livelihood, raising cows, goats, sheep, and, in some families, camels. Additionally, Samburu County is in a global hotspot for climate change vulnerability. Many Samburu families practice polygyny, with up to 5 wives. Gender inequality is substantial, with husbands controlling wives' labor and access to key elements of subsistence (e.g., access to livestock to milk). Women experience high rates of interpersonal spousal violence, which was documented in the overall study.
Recruitment	Samburu live in dispersed homesteads across a rugged landscape, with many homesteads accessible only off road or on foot. The drought catchment area consisted of all rural communities within multiple subregions of Samburu County in which drought effects on livestock mortality were documented by drought monitoring agencies, including lower rainfall areas of the Leroghi Plateau. Recruitment efforts extended to both remote and more accessible communities within those subregions to avoid proximity bias. Recruitment occurred through off road 4WD vehicle and motorcycle transport, reaching all communities within the catchment area at meetings advertised in advance by local village administrators. 123 families were initially recruited, of whom, 11 were found not to meet inclusion criteria at recruitment and 1 family met most criteria but the mother had spent the majority of the pregnancy in Nairobi. Of the 111 families meeting all criteria, 4 families declined to participate, and 3 families agreed but were unavailable during the measurement and biospecimen visit due to long distance herding to follow pasture. No self-selection bias was evident, as all families with individuals who had been pregnant during the drought attended the meetings and were recruited. The number of families declining (4) was 3.6% and did not differ by any known socioeconomic or other characteristics.
Ethics oversight	National Commission for Science, Technology and Innovation (NACOSTI) License [NACOSTI/P/18/7558/22142; P/19/7558/30004]; Western Michigan University for HSIRB [Protocol #17-05-09]; collaborative with University of Nairobi. Site approval was obtained at the Samburu County and local community level, and Samburu community leaders and study partners were collaboratively engaged at all phases of the study. The study was initiated prior to 2018 IRB rules and is subject to reconsent based on individual subject and Indigenous community agreements.

Note that full information on the approval of the study protocol must also be provided in the manuscript.

Field-specific reporting

Please select the one below that is the best fit for your research. If you are not sure, read the appropriate sections before making your selection.

☐ Life sciences ☒ Behavioural & social sciences ☐ Ecological, evolutionary & environmental sciences

For a reference copy of the document with all sections, see nature.com/documents/nr-reporting-summary-flat.pdf

Life sciences study design

All studies must disclose on these points even when the disclosure is negative.

Sample size	*Describe how sample size was determined, detailing any statistical methods used to predetermine sample size OR if no sample-size calculation was performed, describe how sample sizes were chosen and provide a rationale for why these sample sizes are sufficient.*
Data exclusions	*Describe any data exclusions. If no data were excluded from the analyses, state so OR if data were excluded, describe the exclusions and the rationale behind them, indicating whether exclusion criteria were pre-established.*
Replication	*Describe the measures taken to verify the reproducibility of the experimental findings. If all attempts at replication were successful, confirm this OR if there are any findings that were not replicated or cannot be reproduced, note this and describe why.*
Randomization	*Describe how samples/organisms/participants were allocated into experimental groups. If allocation was not random, describe how covariates were controlled OR if this is not relevant to your study, explain why.*
Blinding	*Describe whether the investigators were blinded to group allocation during data collection and/or analysis. If blinding was not possible, describe why OR explain why blinding was not relevant to your study.*

Behavioural & social sciences study design

All studies must disclose on these points even when the disclosure is negative.

Study description	The study employed mixed quantitative and qualitative methods, utilizing a same-sex sibling group design (exposed/unexposed in utero) to collect data based on the natural observational experiment of a severe drought occurring in 2008-2009 in northern Kenya.
Research sample	Samburu pastoralist families exposed to the drought were recruited. The sample is comprised of 104 mothers exposed to the drought during pregnancy, their 104 live born offspring of those pregnancies, and 109 offspring of those mothers who are the same-sex siblings of drought-exposed offspring (child ages 1.81 - 9.61 years). The sample is representative: all families meeting the criteria in the catchment area were included and the number of families declining was small (94% of 111 families meeting criteria participated, with only 4 declining & 3 unavailable - 6%). The sample was selected based on a rigorous same-sex sibling design, and the community selected based on authors' established working rapport and trust with the Indigenous community and their established exposure to severe drought.
Sampling strategy	The study relied on purposive sampling, recruiting throughout rural areas of the dispersed, semi-nomadic target county affected by the 2008-2009 severe drought. All individuals who met the criteria for the same-sex sibling design who agreed to participate were enrolled. The target was a minimum of 100 sibling pairs, based on similar studies (Cao-Lei, Lei, Renaud Massart, Matthew J. Suderman, Ziv Machnes, Guillaume Elgbeili, David P. Laplante, Moshe Szyf, Suzanne King. 2014. DNA Methylation Signatures Triggered by Prenatal Maternal Stress Exposure to a Natural Disaster: Project Ice Storm. PLoS ONE 9(9): e107653; Heijmans, Bastiaan T., Elmar W. Tobi, Aryeh D. Stein, Hein Putter, Gerard J. Blauw, Ezra S. Susser, P.Eline Slagboom, L.H. Lumey. 2008. Persistent Epigenetic Differences Associated with Prenatal Exposure to Famine in Humans. Proceedings of the National Academy of Sciences 105(44): 17046-17049; Tobi, Elmar W., Jelle J. Goeman, Ramin Monajemi, Hongcang Gu, Hein Putter, Yanju Zhang, Roderick C. Slieker, Arthur P. Stok, Peter E. Thijssen, Fabian Müller, Erik W. van Zwet, Christoph Bock, Alexander Meissner, L.H. Lumey, P. Eline Slagboom & Bastiaan T. Heijmans. 2014. DNA Methylation Signatures Link Prenatal Famine Exposure to Growth and Metabolism. Nature Communications 5:5592 doi: 10.1038/ncomms6592).
Data collection	Study team members at the field site consisted of two senior data collection team members, 4 multilingual Samburu research assistants, and up to 3 Kenyan and U.S. undergraduate and graduate students at a time. Team members visited Samburu study partners at locations convenient to study partners (usually their homes). To enhance accuracy and rapport, team members were assigned tasks that they exclusively performed (e.g., anthropometric measurements, ethnographic interviews with mothers, behavioral observations, etc.) All observations and interviews were recorded with pen and paper, with paired team members to cross-check each other's work during data collection. Saliva samples for DNA methylation data were obtained using Oragene 600 kits. Researchers were not blind to hypotheses.
Timing	Rolling recruitment was initiated in October, 2017 and concluded in December 2018. Data collection occurred alongside recruitment and concluded in July 2019.
Data exclusions	No data were excluded from analyses.
Non-participation	4 families declined. Consistent with ethical protocol, reasons were not obtained. 3 families were unavailable during biospecimen collection due to following their herds. Data was collected from 94% of eligible families.
Randomization	In this natural experiment study, children exposed to drought = the experimental group and their same-sex unexposed siblings = control group.

Ecological, evolutionary & environmental sciences study design

All studies must disclose on these points even when the disclosure is negative.

Study description	*Briefly describe the study. For quantitative data include treatment factors and interactions, design structure (e.g. factorial, nested, hierarchical), nature and number of experimental units and replicates.*
Research sample	*Describe the research sample (e.g. a group of tagged Passer domesticus, all Stenocereus thurberi within Organ Pipe Cactus National Monument), and provide a rationale for the sample choice. When relevant, describe the organism taxa, source, sex, age range and any manipulations. State what population the sample is meant to represent when applicable. For studies involving existing datasets, describe the data and its source.*
Sampling strategy	*Note the sampling procedure. Describe the statistical methods that were used to predetermine sample size OR if no sample-size calculation was performed, describe how sample sizes were chosen and provide a rationale for why these sample sizes are sufficient.*
Data collection	*Describe the data collection procedure, including who recorded the data and how.*
Timing and spatial scale	*Indicate the start and stop dates of data collection, noting the frequency and periodicity of sampling and providing a rationale for these choices. If there is a gap between collection periods, state the dates for each sample cohort. Specify the spatial scale from which the data are taken*
Data exclusions	*If no data were excluded from the analyses, state so OR if data were excluded, describe the exclusions and the rationale behind them, indicating whether exclusion criteria were pre-established.*
Reproducibility	*Describe the measures taken to verify the reproducibility of experimental findings. For each experiment, note whether any attempts to repeat the experiment failed OR state that all attempts to repeat the experiment were successful.*
Randomization	*Describe how samples/organisms/participants were allocated into groups. If allocation was not random, describe how covariates were controlled. If this is not relevant to your study, explain why.*
Blinding	*Describe the extent of blinding used during data acquisition and analysis. If blinding was not possible, describe why OR explain why blinding was not relevant to your study.*

Did the study involve field work? ☐ Yes ☐ No

Field work, collection and transport

Field conditions	*Describe the study conditions for field work, providing relevant parameters (e.g. temperature, rainfall).*
Location	*State the location of the sampling or experiment, providing relevant parameters (e.g. latitude and longitude, elevation, water depth).*
Access & import/export	*Describe the efforts you have made to access habitats and to collect and import/export your samples in a responsible manner and in compliance with local, national and international laws, noting any permits that were obtained (give the name of the issuing authority, the date of issue, and any identifying information).*
Disturbance	*Describe any disturbance caused by the study and how it was minimized.*

Reporting for specific materials, systems and methods

We require information from authors about some types of materials, experimental systems and methods used in many studies. Here, indicate whether each material, system or method listed is relevant to your study. If you are not sure if a list item applies to your research, read the appropriate section before selecting a response.

Materials & experimental systems

n/a	Involved in the study	
☒	☐	Antibodies
☒	☐	Eukaryotic cell lines
☒	☐	Palaeontology and archaeology
☒	☐	Animals and other organisms
☒	☐	Clinical data
☒	☐	Dual use research of concern
☒	☐	Plants

Methods

n/a	Involved in the study	
☒	☐	ChIP-seq
☒	☐	Flow cytometry
☒	☐	MRI-based neuroimaging

Antibodies

Antibodies used | *Describe all antibodies used in the study; as applicable, provide supplier name, catalog number, clone name, and lot number.*

Validation | *Describe the validation of each primary antibody for the species and application, noting any validation statements on the manufacturer's website, relevant citations, antibody profiles in online databases, or data provided in the manuscript.*

Eukaryotic cell lines

Policy information about cell lines and Sex and Gender in Research

Cell line source(s) | *State the source of each cell line used and the sex of all primary cell lines and cells derived from human participants or vertebrate models.*

Authentication | *Describe the authentication procedures for each cell line used OR declare that none of the cell lines used were authenticated.*

Mycoplasma contamination | *Confirm that all cell lines tested negative for mycoplasma contamination OR describe the results of the testing for mycoplasma contamination OR declare that the cell lines were not tested for mycoplasma contamination.*

Commonly misidentified lines (See ICLAC register) | *Name any commonly misidentified cell lines used in the study and provide a rationale for their use.*

Palaeontology and Archaeology

Specimen provenance | *Provide provenance information for specimens and describe permits that were obtained for the work (including the name of the issuing authority, the date of issue, and any identifying information). Permits should encompass collection and, where applicable, export.*

Specimen deposition | *Indicate where the specimens have been deposited to permit free access by other researchers.*

Dating methods | *If new dates are provided, describe how they were obtained (e.g. collection, storage, sample pretreatment and measurement), where they were obtained (i.e. lab name), the calibration program and the protocol for quality assurance OR state that no new dates are provided.*

☐ Tick this box to confirm that the raw and calibrated dates are available in the paper or in Supplementary Information.

Ethics oversight | *Identify the organization(s) that approved or provided guidance on the study protocol, OR state that no ethical approval or guidance was required and explain why not.*

Note that full information on the approval of the study protocol must also be provided in the manuscript.

Animals and other research organisms

Policy information about studies involving animals; ARRIVE guidelines recommended for reporting animal research, and Sex and Gender in Research

Laboratory animals | *For laboratory animals, report species, strain and age OR state that the study did not involve laboratory animals.*

Wild animals | *Provide details on animals observed in or captured in the field; report species and age where possible. Describe how animals were caught and transported and what happened to captive animals after the study (if killed, explain why and describe method; if released, say where and when) OR state that the study did not involve wild animals.*

Reporting on sex | *Indicate if findings apply to only one sex; describe whether sex was considered in study design, methods used for assigning sex. Provide data disaggregated for sex where this information has been collected in the source data as appropriate; provide overall numbers in this Reporting Summary. Please state if this information has not been collected. Report sex-based analyses where performed, justify reasons for lack of sex-based analysis.*

Field-collected samples | *For laboratory work with field-collected samples, describe all relevant parameters such as housing, maintenance, temperature, photoperiod and end-of-experiment protocol OR state that the study did not involve samples collected from the field.*

Ethics oversight | *Identify the organization(s) that approved or provided guidance on the study protocol, OR state that no ethical approval or guidance was required and explain why not.*

Note that full information on the approval of the study protocol must also be provided in the manuscript.

Clinical data

Policy information about clinical studies

All manuscripts should comply with the ICMJE guidelines for publication of clinical research and a completed CONSORT checklist must be included with all submissions.

Clinical trial registration	*Provide the trial registration number from ClinicalTrials.gov or an equivalent agency.*
Study protocol	*Note where the full trial protocol can be accessed OR if not available, explain why.*
Data collection	*Describe the settings and locales of data collection, noting the time periods of recruitment and data collection.*
Outcomes	*Describe how you pre-defined primary and secondary outcome measures and how you assessed these measures.*

Dual use research of concern

Policy information about dual use research of concern

Hazards

Could the accidental, deliberate or reckless misuse of agents or technologies generated in the work, or the application of information presented in the manuscript, pose a threat to:

No	Yes	
☐	☐	Public health
☐	☐	National security
☐	☐	Crops and/or livestock
☐	☐	Ecosystems
☐	☐	Any other significant area

Experiments of concern

Does the work involve any of these experiments of concern:

No	Yes	
☐	☐	Demonstrate how to render a vaccine ineffective
☐	☐	Confer resistance to therapeutically useful antibiotics or antiviral agents
☐	☐	Enhance the virulence of a pathogen or render a nonpathogen virulent
☐	☐	Increase transmissibility of a pathogen
☐	☐	Alter the host range of a pathogen
☐	☐	Enable evasion of diagnostic/detection modalities
☐	☐	Enable the weaponization of a biological agent or toxin
☐	☐	Any other potentially harmful combination of experiments and agents

Plants

Seed stocks	*Report on the source of all seed stocks or other plant material used. If applicable, state the seed stock centre and catalogue number. If plant specimens were collected from the field, describe the collection location, date and sampling procedures.*
Novel plant genotypes	*Describe the methods by which all novel plant genotypes were produced. This includes those generated by transgenic approaches, gene editing, chemical/radiation-based mutagenesis and hybridization. For transgenic lines, describe the transformation method, the number of independent lines analyzed and the generation upon which experiments were performed. For gene-edited lines, describe the editor used, the endogenous sequence targeted for editing, the targeting guide RNA sequence (if applicable) and how the editor was applied.*
Authentication	*Describe any authentication procedures for each seed stock used or novel genotype generated. Describe any experiments used to assess the effect of a mutation and, where applicable, how potential secondary effects (e.g. second site T-DNA insertions, mosiacism, off-target gene editing) were examined.*

ChIP-seq

Data deposition

- [ ] Confirm that both raw and final processed data have been deposited in a public database such as GEO.
- [ ] Confirm that you have deposited or provided access to graph files (e.g. BED files) for the called peaks.

Data access links<br>*May remain private before publication.*	*For "Initial submission" or "Revised version" documents, provide reviewer access links. For your "Final submission" document, provide a link to the deposited data.*
Files in database submission	*Provide a list of all files available in the database submission.*
Genome browser session<br>(e.g. UCSC)	*Provide a link to an anonymized genome browser session for "Initial submission" and "Revised version" documents only, to enable peer review. Write "no longer applicable" for "Final submission" documents.*

Methodology

Replicates	*Describe the experimental replicates, specifying number, type and replicate agreement.*
Sequencing depth	*Describe the sequencing depth for each experiment, providing the total number of reads, uniquely mapped reads, length of reads and whether they were paired- or single-end.*
Antibodies	*Describe the antibodies used for the ChIP-seq experiments; as applicable, provide supplier name, catalog number, clone name, and lot number.*
Peak calling parameters	*Specify the command line program and parameters used for read mapping and peak calling, including the ChIP, control and index files used.*
Data quality	*Describe the methods used to ensure data quality in full detail, including how many peaks are at FDR 5% and above 5-fold enrichment.*
Software	*Describe the software used to collect and analyze the ChIP-seq data. For custom code that has been deposited into a community repository, provide accession details.*

Flow Cytometry

Plots

Confirm that:

- [ ] The axis labels state the marker and fluorochrome used (e.g. CD4-FITC).
- [ ] The axis scales are clearly visible. Include numbers along axes only for bottom left plot of group (a 'group' is an analysis of identical markers).
- [ ] All plots are contour plots with outliers or pseudocolor plots.
- [ ] A numerical value for number of cells or percentage (with statistics) is provided.

Methodology

Sample preparation	*Describe the sample preparation, detailing the biological source of the cells and any tissue processing steps used.*
Instrument	*Identify the instrument used for data collection, specifying make and model number.*
Software	*Describe the software used to collect and analyze the flow cytometry data. For custom code that has been deposited into a community repository, provide accession details.*
Cell population abundance	*Describe the abundance of the relevant cell populations within post-sort fractions, providing details on the purity of the samples and how it was determined.*
Gating strategy	*Describe the gating strategy used for all relevant experiments, specifying the preliminary FSC/SSC gates of the starting cell population, indicating where boundaries between "positive" and "negative" staining cell populations are defined.*

- [ ] Tick this box to confirm that a figure exemplifying the gating strategy is provided in the Supplementary Information.

Magnetic resonance imaging

Experimental design

Design type	*Indicate task or resting state; event-related or block design.*

Design specifications | *Specify the number of blocks, trials or experimental units per session and/or subject, and specify the length of each trial or block (if trials are blocked) and interval between trials.*

Behavioral performance measures | *State number and/or type of variables recorded (e.g. correct button press, response time) and what statistics were used to establish that the subjects were performing the task as expected (e.g. mean, range, and/or standard deviation across subjects).*

Acquisition

Imaging type(s) | *Specify: functional, structural, diffusion, perfusion.*

Field strength | *Specify in Tesla*

Sequence & imaging parameters | *Specify the pulse sequence type (gradient echo, spin echo, etc.), imaging type (EPI, spiral, etc.), field of view, matrix size, slice thickness, orientation and TE/TR/flip angle.*

Area of acquisition | *State whether a whole brain scan was used OR define the area of acquisition, describing how the region was determined.*

Diffusion MRI ☐ Used ☐ Not used

Preprocessing

Preprocessing software | *Provide detail on software version and revision number and on specific parameters (model/functions, brain extraction, segmentation, smoothing kernel size, etc.).*

Normalization | *If data were normalized/standardized, describe the approach(es): specify linear or non-linear and define image types used for transformation OR indicate that data were not normalized and explain rationale for lack of normalization.*

Normalization template | *Describe the template used for normalization/transformation, specifying subject space or group standardized space (e.g. original Talairach, MNI305, ICBM152) OR indicate that the data were not normalized.*

Noise and artifact removal | *Describe your procedure(s) for artifact and structured noise removal, specifying motion parameters, tissue signals and physiological signals (heart rate, respiration).*

Volume censoring | *Define your software and/or method and criteria for volume censoring, and state the extent of such censoring.*

Statistical modeling & inference

Model type and settings | *Specify type (mass univariate, multivariate, RSA, predictive, etc.) and describe essential details of the model at the first and second levels (e.g. fixed, random or mixed effects; drift or auto-correlation).*

Effect(s) tested | *Define precise effect in terms of the task or stimulus conditions instead of psychological concepts and indicate whether ANOVA or factorial designs were used.*

Specify type of analysis: ☐ Whole brain ☐ ROI-based ☐ Both

Statistic type for inference | *Specify voxel-wise or cluster-wise and report all relevant parameters for cluster-wise methods.*

(See Eklund et al. 2016)

Correction | *Describe the type of correction and how it is obtained for multiple comparisons (e.g. FWE, FDR, permutation or Monte Carlo).*

Models & analysis

n/a	Involved in the study
☐	☐ Functional and/or effective connectivity
☐	☐ Graph analysis
☐	☐ Multivariate modeling or predictive analysis

Functional and/or effective connectivity | *Report the measures of dependence used and the model details (e.g. Pearson correlation, partial correlation, mutual information).*

Graph analysis | *Report the dependent variable and connectivity measure, specifying weighted graph or binarized graph, subject- or group-level, and the global and/or node summaries used (e.g. clustering coefficient, efficiency, etc.).*

Multivariate modeling and predictive analysis | *Specify independent variables, features extraction and dimension reduction, model, training and evaluation metrics.*
